# Augmenting regulatory T cells: new therapeutic strategy for rheumatoid arthritis

**DOI:** 10.3389/fimmu.2024.1312919

**Published:** 2024-01-23

**Authors:** Jiaqian Zhang, Hongjiang Liu, Yuehong Chen, Huan Liu, Shengxiao Zhang, Geng Yin, Qibing Xie

**Affiliations:** ^1^ Department of Rheumatology and Immunology, West China Hospital, Sichuan University, Chengdu, China; ^2^ Department of Rheumatology, The Second Hospital of Shanxi Medical University, Taiyuan, China; ^3^ Department of General Practice, General Practice Medical Center, West China Hospital, Sichuan University, Chengdu, China

**Keywords:** regulatory T cells, rheumatoid arthritis, therapy, Foxp3, Th17

## Abstract

Rheumatoid arthritis (RA) is a chronic, systemic autoimmune condition marked by inflammation of the joints, degradation of the articular cartilage, and bone resorption. Recent studies found the absolute and relative decreases in circulating regulatory T cells (Tregs) in RA patients. Tregs are a unique type of cells exhibiting immunosuppressive functions, known for expressing the Foxp3 gene. They are instrumental in maintaining immunological tolerance and preventing autoimmunity. Increasing the absolute number and/or enhancing the function of Tregs are effective strategies for treating RA. This article reviews the studies on the mechanisms and targeted therapies related to Tregs in RA, with a view to provide better ideas for the treatment of RA.

## 1 Introduction

Rheumatoid arthritis (RA) is a chronic inflammatory disease characterized by symmetric arthritis in distal joints, leading to joint degradation and functional impairment. It often presents with extra-articular manifestations, involving multiple systems, severely impacting overall bodily function and reducing the quality of life for patients (1). Nonsteroidal anti-inflammatory drugs, glucocorticoids, and rheumatoid arthritis medications are commonly used to treat RA to alleviate inflammation and reduce pain. However, the use of these drugs carries the risk of liver, kidney, and gastrointestinal damage (2). The emergence of early diagnosis, novel treatment methods, and effective therapeutic strategies has garnered widespread attention. Currently, the etiology of RA is not fully elucidated, but the immune processes occurring in the synovium and synovial fluid, including synovial cell proliferation and fibrosis, vascular membrane formation, and cartilage and bone erosion, are noteworthy. Naive CD4+ T cells can differentiate into various cell types under antigen-presenting cell stimulation. Dysregulation in the function and/or quantity of these cells can lead to abnormalities in cellular and humoral immunity (3). Regulatory T cells (Tregs) play a crucial role in maintaining immune tolerance and preventing autoimmunity (4, 5). Abnormal quantity and/or functional deficiencies in Tregs have been reported in RA (6–9). Therefore, manipulating and regulating Tregs represent effective therapeutic strategies for many such diseases.

## 2 Characteristics and functions of Tregs

### 2.1 Origin and classification of Tregs

Tregs, initially identified in animal models and subsequently in humans, have offered a novel viewpoint on the establishment and preservation of self-tolerance (4). In 1995, Sakaguchi first discovered a CD4^+^CD25^+^ T cell subset with autoimmune tolerance capability further named Tregs (10). In 2003, forkhead box protein 3 (Foxp3) was established as a critical transcription factor for Tregs, indispensable for their formation and suppressive functionality. Considering their origin and biological traits, Tregs are classified into two subgroups: thymus-derived regulatory T cells (tTregs), also referred to as natural regulatory T cells (nTregs), and induced regulatory T cells (iTregs), or peripheral regulatory T cells (pTregs). Despite significant differences in origin, gene expression profiles, and biological characteristics between nTregs and iTregs (11), both types are dependent on antigen stimulation and activation in the presence of IL-2 (12). nTregs are strictly controlled by the thymic microenvironment and characteristically express the nuclear transcription factor Foxp3, along with CD25 and CD4 on their cell surface. Induced Tregs, or iTregs, primarily originate from immature CD4^+^ cells in peripheral lymphoid tissues or from *in vitro* culture, stimulated by transforming growth factor (TGF)-β and IL-2. The induction of iTregs does not require strong T cell receptor (TCR) stimulation, but rather an appropriate antigen stimulation. Overactivation of downstream TCR signals may interfere with iTreg development. Similarly, strong co-stimulation negatively impacts the generation of iTregs. Conversely, blocking the CD28 co-stimulatory pathway or improving signal transduction through the activation of cytotoxic T lymphocyte-associated protein 4 (CTLA4) and ICOS promotes the development of iTregs (13). Although both nTregs and iTregs express Foxp3, CD25, CTLA-4, and other Treg function-related molecules, the stability of Foxp3 expression in iTregs is much lower than in nTregs. Under certain *in vivo* conditions, iTregs can be driven to differentiate into effector T cells (14). In nTregs, the DNA in the regulatory T cell-specific demethylated region (TSDR) of the Foxp3 enhancer is demethylated, while in iTregs, the TSDR is only partially demethylated (15). This incomplete demethylation renders iTregs more susceptible to loss of Foxp3 expression and functionality. nTregs represent a stable lineage of cells specific to antigens expressed in the thymus, whereas iTregs constitute a more dynamic population, ensuring tolerance to new antigens encountered in peripheral areas. Collectively, these two populations are vital for maintaining immune tolerance.

### 2.2 Structure and function of Foxp3

Foxp3, located on the p11.23-13.3 region of the X chromosome, consists of 11 coding exons, 3 non-coding exons, and 104 introns. It serves as the main transcription factor dictating the development and function of Tregs (16). Within the Foxp3 locus, there are three conserved non-coding sequences (CNSs): a promoter (CNS1) and two enhancers (CNS2, CNS3) (17). CNS1 is essential for the induction of peripheral Tregs, and its knockout significantly reduces the number of Tregs in gut-associated lymphoid tissues (18). CNS3 plays a crucial role in the genesis of Tregs, and its deletion leads to a severe reduction in the thymic output of Tregs. CNS2, also known as TSDR, contains a CpG island that is highly demethylated only in functional Tregs. The methylation status of this highly conserved CpG island is pivotal in determining the expression level of Foxp3 and the stability of Tregs, and is deemed the most definitive marker of the Treg lineage (18). Two primary epigenetic mechanisms: DNA methylation and histone modifications play crucial roles in establishing and maintaining Foxp3 expression in Tregs (19, 20). Methylation of the TSDR can lead to chromatin condensation, which decreases the DNA sequence’s accessibility and inhibits the transcription of Foxp3. The partial methylation of TSDR underlies the instability of Foxp3 expression in Tregs. Conversely, the demethylation of TSDR is vital for the stability of Foxp3 expression, promoting transcription in Tregs. Moreover, Foxp3 is also regulated by ubiquitination and phosphorylation processes. Ubiquitination encourages Foxp3 degradation, while the impacts of Foxp3 phosphorylation can vary depending on the modification site. Protein phosphatase 1 acts on Foxp3 at the Ser418 site, removing a phosphate group - a process known as dephosphorylation. On the other hand, PIM1 adds a phosphate group to the Ser422 site of Foxp3, a process known as phosphorylation, which inhibits the function of Foxp3. In a contrasting manner, the lymphocyte-specific protein tyrosine kinase phosphorylates Foxp3 at the Tyr342 site, which augments its function. Therefore, the expression of Foxp3 must be strictly regulated to maintain the homeostasis of T cell-mediated immune responses. Tregs expressing Foxp3 play a significant role in suppressing immune responses (21). Constitutive expression of Foxp3 is a decisive factor in driving immune-suppressive functions (16). Mice with Foxp3 defects develop fatal autoimmune diseases (22), whereas lifelong continuous expression of Foxp3 prevents the occurrence of autoimmunity (23). Tregs with downregulated Foxp3 expression lose their suppressive function and can even produce pro-inflammatory cytokines, such as effector cells (24). Therefore, the stable expression of Foxp3 plays an important role in the balance between autoimmunity and tolerance, as well as the therapeutic effect based on Tregs.

### 2.3 Immunosuppressive mechanism of Tregs

**Figure 1:**
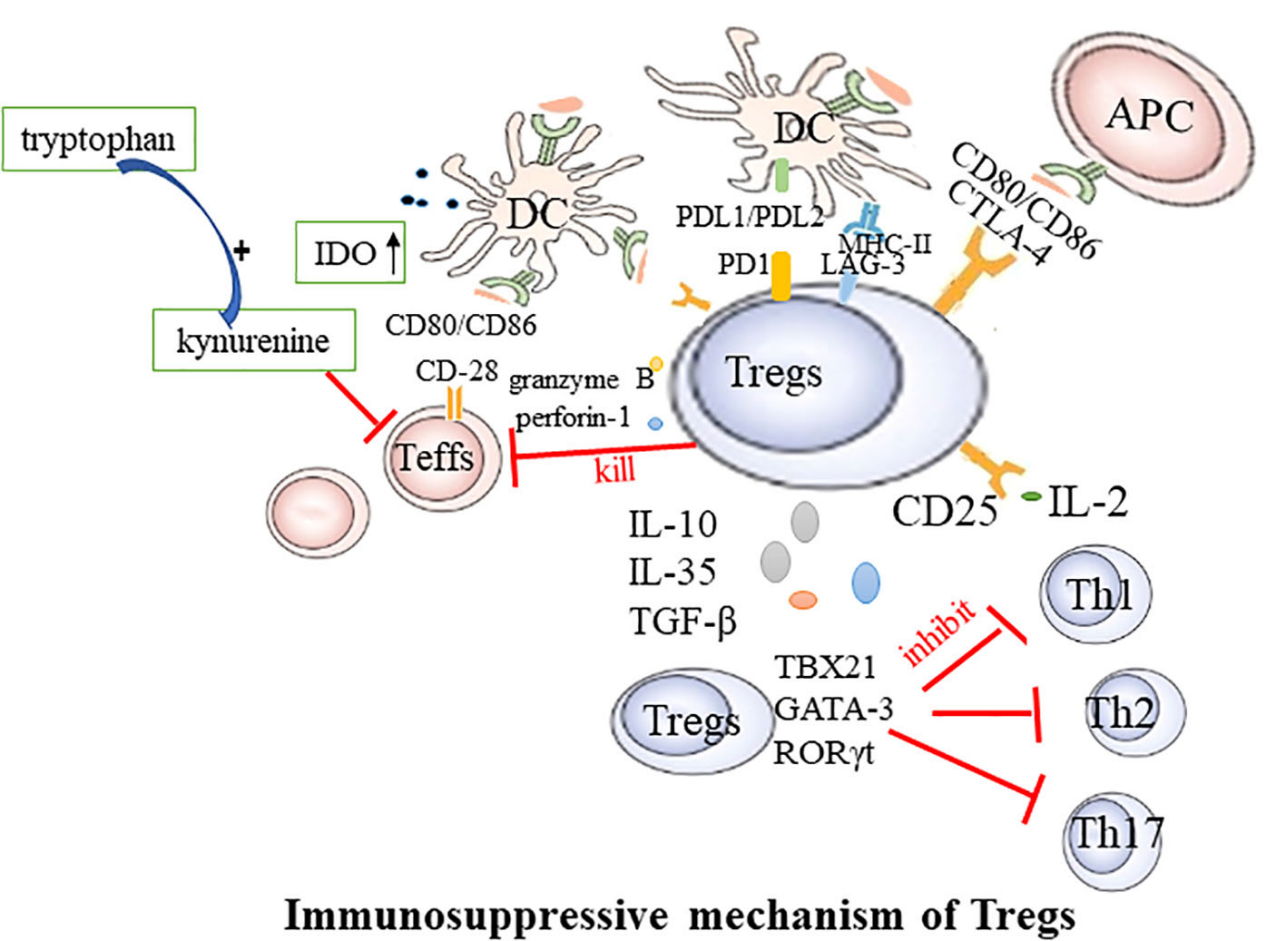
Immunosuppressive mechanism of Tregs. Teffs, effector T cells; DC, dendritic cells; CTLA-4, Cytotoxic T lymphocyte-associated antigen4; LAG-3, Lymphocyte activation gene 3; PD-1, Programmed cell death protein 1; APC, antigen presenting cells; TBX21, T-box transcription factor; Th, T helper cell.

RA is a systemic, inflammatory autoimmune disease. Aberrant activation of innate immune cells, including dendritic cells (DCs), innate lymphoid cells, and adaptive immune cells, may lead to excessive production of pro-inflammatory cytokines, ultimately resulting in the destruction of bone tissue and cartilage (25).Tregs play an irreplaceable role in maintaining self-tolerance and homeostasis, presenting noticeable differences between healthy individuals and those with autoimmune diseases (26, 27). They regulate the immune response through intercellular interactions or by inhibiting cytokine release and inducing negative regulatory signaling molecules to suppress excessive immunity. The potential mechanisms through which Tregs mediate inhibition are as follows: (**Figure 1**).

### 2.3.1 Cell-to-cell contact-dependent

Tregs exercise their suppressive function via direct engagement with DCs or effector T cells (Teffs). This regulation of immune responses is facilitated through a mechanism that relies on cell-to-cell contact and is characterized by the expression of the nuclear transcription factor Foxp3, in conjunction with the elevated presence of CD25, CTLA4, Lymphocyte activation gene 3 (LAG-3), and glucocorticoid-induced TNFR-related protein (GITR) on the cell membrane. CTLA-4, an inhibitory molecule, is abundantly expressed on the Tregs’ surface and is instrumental in their function. Tregs with CTLA-4 deficiency exhibit significantly weakened suppressive effects (28). CTLA-4 curbs T-cell activation by either competitively binding to or downregulating CD80/CD86 present on the surface of DCs (29, 30). Additionally, Tregs can deplete CD80 and CD86 from the surface of antigen-presenting cells (APCs) through a process of endocytosis mediated by CTLA-4, thereby further modulating the stimulatory capacity of APCs (31). CTLA-4 can also induce indoleamine 2,3-dioxygenase (IDO) in dendritic cells. IDO degrades tryptophan into the immunotoxic metabolite kynurenine, inhibiting the proliferation of Teffs. LAG-3 mitigates the costimulatory capacity of dendritic cells (DCs) and the immune response of T cells by binding to MHC-II (32). The surface Programmed Cell Death Protein 1(PD-1), also known as CD279, on Tregs binds to its ligands PD-L1 and PD-L2 on DCs, leading to the inhibition of effector T cell function (33). Additionally, Foxp3^+^ Tregs expressing the transcription factors TBX21, GATA-3, or retinoic acid receptor-related orphan receptor (ROR) γt can respectively suppress the functions of helper T cells 1 (Th1), 2 (Th2), or 17 (Th17) (34).

### 2.3.2 Cell contact-independent

Tregs wield a potent immunosuppressive influence on the proliferation and cytokine production of effector T cells. By depleting and generating key cytokines, Tregs regulate immune responses and the maintenance of immune homeostasis. The high level of CD25 expression on the surface of Tregs contributes to the massive consumption of IL-2. This cytokine is required for Teffs proliferation and activation. Therefore, Tregs may competitively deplete this growth factor from pathogenic Teffs, thereby indirectly inhibiting their activity (35). The suppressive effects of Tregs may also occur through the secretion of soluble immunosuppressive cytokines, including IL-10, TGF-β, and IL-35 (36). These cytokines further contribute to the immunomodulatory effects of Tregs. For example, B-lymphocyte-induced maturation protein 1 promotes the differentiation of Tregs, upregulates IL-10, and inhibits CD4+ T cell-induced activation of fibroblast-like synoviocytes (37). In mouse models of collagen-induced arthritis and inflammatory bowel disease, Tregs lacking IL-35 are unable to control disease progression, highlighting the importance of this cytokine in immune regulation (38). Serum IL-35 levels are reduced in rheumatoid arthritis (RA) patients, demonstrating potential as a biomarker for active RA (39). Tregs can kill responsive T cells through the secretion of granzyme B and perforin-1 (40). In addition to their local effects, Tregs can “sense” cytokines or chemokines in the environment and migrate to sites of inflammation. During the active immune response, stimulation of TCR and cytokines guide Tregs to migrate to the site of inflammation. Once there, they suppress the inflammatory response, limit tissue damage, and promote resolution of inflammation (41).

## 3 Characteristics of Tregs in RA

Under normal circumstances, Tregs regulate immune tolerance status. Studies have shown that the occurrence and development of RA are related to various changes in anti-inflammatory Tregs and their counter-regulatory effects. Abnormal number and functional defects of Tregs play a relevant role in the pathogenesis and development of RA (7, 9, 42).

### 3.1 Deficiency of Tregs in RA

A deficiency of Tregs has been observed in both RA animal models and patients. In collagen-induced arthritis (CIA), a commonly used model for RA that involves immunizing mice with heterologous CII, an abnormal number of Tregs has been shown to influence the disease. In CIA mice, increased Treg counts were closely associated with reduced joint swelling, lower arthritis scores, reduced cartilage surface damage, and reduced joint cavity desmoplasia (43). Transfer of CD4^+^CD25^+^ cells into immunized mice during induction of antigen-induced arthritis resulted in reduced arthritis severity. Effective depletion of Tregs at any point in the life of a healthy individual triggers a rapid autoimmune and inflammatory response, resulting in multi-organ autoimmunity (44). The role of Tregs in pathogenesis is not fully understood, and the number of Tregs reported over the years to be increased, decreased, or unchanged in synovial fluid and peripheral blood is controversial (**Table 1**). The controversy surrounding the reported fluctuations in regulatory T cells (Tregs) numbers in the synovial fluid and peripheral blood of rheumatoid arthritis (RA) patients can be attributed to several factors. Variations in the local immune microenvironment within the joint may contribute to differences observed in Tregs numbers. The heterogeneous nature of the RA patient population, including diverse disease progressions, clinical manifestations, and treatment responses, may lead to inconsistent findings across studies. Methodological disparities, such as differences in detection methods, criteria for defining Tregs and different statistical counting methods (absolute value or percentage of Tregs), further contribute to the discrepancies. Therapeutic interventions, ranging from immunosuppressive agents to anti-inflammatory medications, may impact Tregs numbers and function in RA patients. Additionally, the variability in sample collection timing and individual differences, including genetic variations, play roles in shaping the observed differences in Tregs numbers among RA patients. Therefore, a comprehensive understanding of Tregs’ role in RA necessitates consideration of study design, sample sources, detection methods, and other influencing factors. Although the proportion of Tregs in the peripheral blood of RA patients is reported differently, most studies consistently report an abundance of Tregs in SF. The low frequency of Tregs in the peripheral blood of RA patients may be due to these cells migrating to local inflammatory tissues such as synovial fluid by expressing specific chemokine receptors. Some investigations of the absolute number of lymphocyte subsets in RA patients and its clinical significance found that the absolute number of Treg subsets can better reflect the true state of RA patients, making it more clinically significant (64, 67). In patients with rheumatic diseases, the proportion of Tregs varies according to the cell identification method used. A more stringent definition of Tregs is needed when assessing the status of such patients.

**Table 1:** Treg numbers in RA patients.

References	Cell Types	Position	Treg numbers	Years
(45)	CD25^bright^CD4^+^ T cells	SF>PB	increase	2003
(46)	CD25^bright^CD4^+^ T cells	SF	increase	2004
		PB	decrease	
(47–49)	CD4^+^ CD25^+^ T cells	SF>PB	increase	2004-2005
		PB	normal	
(50)	CD4^+^CD25^+^ Tregs	PB	increase	2006
(51)	CD4^+^CD25^+^ Foxp3^+^	SF	increase	2007
		PB	decrease	
(52)	CD4^+^CD25^high^ Tregs	PB	increase	2008
(53)	CD4^+^CD25^high^, CD4^+^CD25^int^, CD4^+^CD25^int/high^FoxP3^+^	PB	decrease	2009
(54)	CD4^+^CD25^high^CD^127low/-^ Tregs	PB	decrease/normal	2011
(55)	CD4^+^CD25^+^ Tregs	PB	decrease	2011
(56)	CD4^+^CD25^high^Foxp3^+^ Tregs	PB	decrease	2011
(57)	CD4^+^CD25^high^ Foxp3^+^ Tregs	PB	decrease	2012
(58)	CD4^+^ Foxp3^+^ T cells	PB	normal	2013
(59)	CD4^+^CD45^RO+^CD25^+^CD^127low^Tregs	PB	normal	2013
		SF>PB	–	
(60)	CD4^+^CD25^^+^/high^CD^127low/-^ Tregs	SM	increase	2014
		SF		
		PB		
(61)	CD3^+^CD4^+^CD25^+^CD^127low^ Tregs	PB	normal	2016
(62)	CD4^+^CD25^+^FoxP3^+^ Tregs	PB	decrease	2016
		SF	increase	
(63)	CD4^+^CD25^high^CD127^−^ Tregs	PB	decrease	2018
(64)	CD4^+^CD25^+^FoxP3^+^ Tregs	refractory RA PB	decrease	2022
(65)	CD4 ^+^ Foxp3^+^Tregs	PB	decrease	2023
(66)	CD4^+^CD25^+^FoxP3^+^ Tregs	early RA PB	normal	2023
		treated RA PB	decrease	

PB, peripheral blood; SF, synovial fluid; SM, Synovial membrane.

### 3.2 Malfunction of Tregs in RA

In the CIA model of Sprague Dawley rats, there is a significant decrease in the expression level of Foxp3, a key transcription factor in Tregs. This finding further underscores the importance of Foxp3 in maintaining immune homeostasis and preventing autoimmune diseases (68). During the pathological process of RA, the levels of pro-inflammatory cytokines in the serum, such as interleukin-6 (IL-6) and tumor necrosis factor-α (TNF-α), are significantly elevated. These factors play a crucial role in the initiation and progression of the disease (69). Through complex signaling pathways, these factors impact the stability of Foxp3 in Tregs, altering their functionality and reducing their efficacy in suppressing immune responses. At the gene transcription level, IL-6 promotes DNA methylation at the CpG site of the upstream enhancer in nTreg, a process that weakens the transcriptional activity of the Foxp3 gene. At the protein level, IL-6 activates the enzyme PIM1, which specifically phosphorylates the S422 site of Foxp3. This process negatively regulates the chromatin binding activity of Foxp3, thereby inhibiting the function of Tregs (70). On the other hand, IL-1β can downregulate Foxp3 expression induced by TGF-β, while TNF-α activates protein phosphatase 1 to dephosphorylate the Ser418 site of Foxp3. Both mechanisms could potentially reduce the suppressive function of Tregs (71, 72). Moreover, inflammatory cytokines can regulate the expression of the Foxp3 transcription factor directly through nuclear factor-κB, thereby affecting the development and function of Tregs (73).

Inflammatory cytokines, beyond their direct regulation of Foxp3, wield an indirect influence on the functionality of Tregs by modulating the behavior of other cellular entities. For instance, a constellation of inflammatory cytokines, including but not limited to IL-1β, IL-6, and TNF-α, orchestrate a complex interplay that indirectly governs the proliferation of Tregs. This regulation occurs through the modulation of DCs, pivotal players in our immune response. The intricate dance of cell signaling also involves IL-21, a cytokine found in conventional T cells. It indirectly maintains the balance - or homeostasis - of Tregs by curtailing the availability of another cytokine, IL-2 (74). Adding another layer to this complex picture, Sun et al. (75) has traced the functional impairments seen in Tregs in the context of RA back to a reduction in the expression of a particular protein - T-cell immunoglobulin and mucin-domain containing-3 (62). In a separate but related discovery, Wang L. and his team found that exosomes derived from RA can inhibit the induction of Tregs. They achieve this by transferring a specific microRNA, miR-17, which subsequently suppresses the differentiation of Tregs by targeting the expression of TGFBR II, a crucial receptor in this process (76). In the battlefield of RA, Tregs are often unable to halt the onslaught of abnormal immune responses and the relentless progression of the disease. The question remains: is this inability linked to an early aging process in Tregs, which could lead to alterations in their phenotype and function? This is still an elusive piece of the puzzle that researchers are striving to understand (77).

### 3.3 Imbalance of Tregs/Teffs in RA

In RA patients, the number and function of peripheral Tregs are defective, leading to excessive activation of Teffs and promoting the onset of RA. This phenomenon is particularly evident during the active phase of RA (57, 68). The expression of Th17 cells, Th1, and Th17-related cytokines, and RORγt in peripheral blood of patients with active RA is increased, and the expression of Tregs and Foxp3 is decreased (78). The etiology and pathogenesis of RA remain unclear, but T cell dysfunction, particularly the abnormal activation of helper CD4^+^T cells, is a primary driver in the occurrence and development of RA (79). These T cells differentiate into different subsets: Th1, Th2, Th17, and Tregs, under the regulation of specific transcription factors (80).

**Figure 2:**
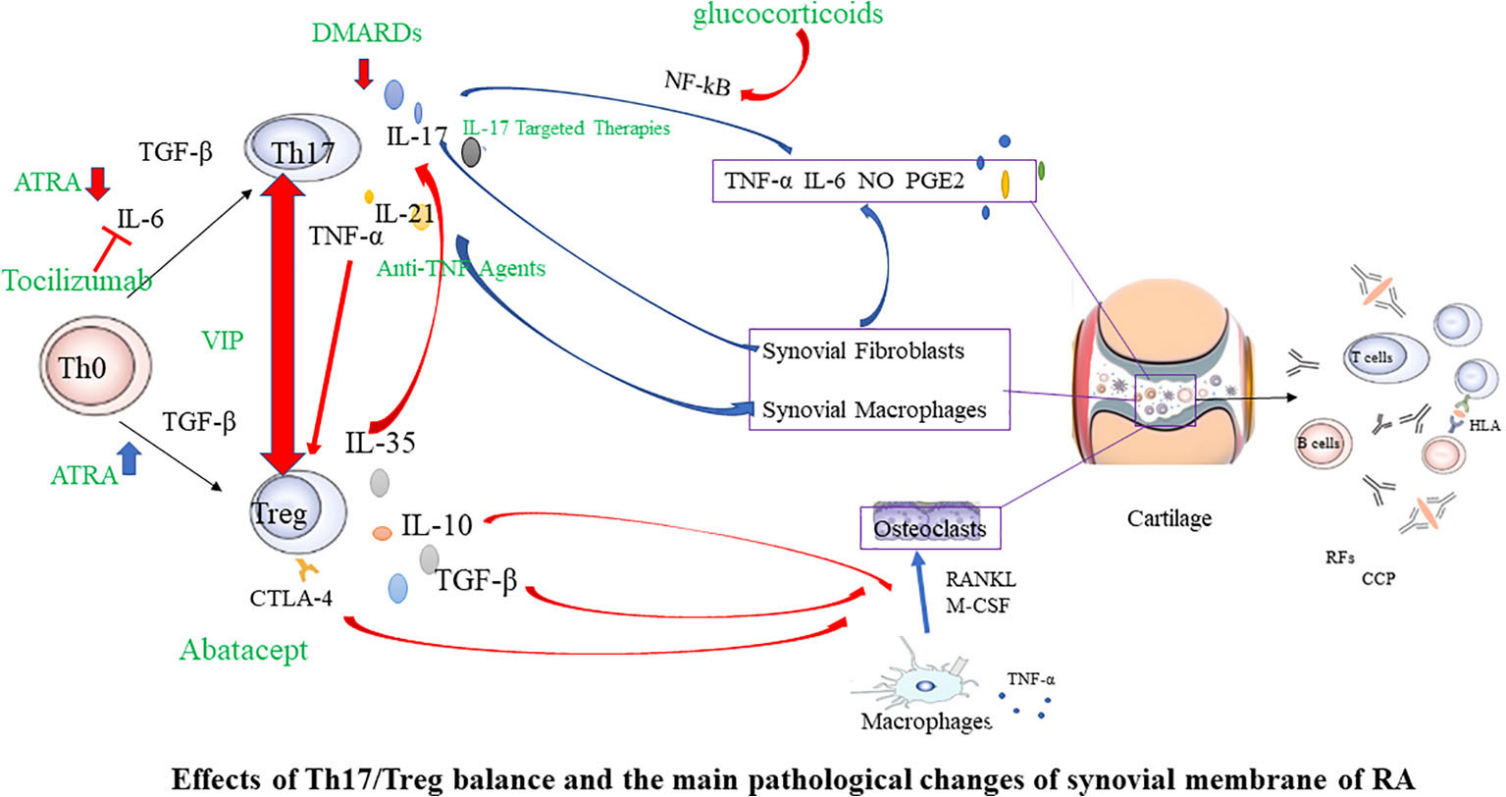
Effects of Th17/Treg balance and the main pathological changes of synovial membrane of RA. In the initial Th0 cells, in the presence of TGF-β, they differentiate into Tregs. However, when IL-6 is present simultaneously, it induces the expression of RORγt, promoting the differentiation into Th17 cells. Tregs have the ability to downregulate the expression of IL-17, thereby inhibiting the differentiation of Th17 cells. Conversely, inhibiting the production of Th17 cells helps promote the development of Tregs. In the joints, synovial cells and chondrocytes are the major local cell populations affected by rheumatoid arthritis. Synovial cells can be classified as fibroblast-like and macrophage-like synoviocytes. In affected joints, synovium swells due to infiltration by fibroblast-like synoviocytes, various T cell subsets, macrophages, and B cells. Interactions among these cells lead to the excessive production of numerous cytokines, sustaining synovial inflammation and joint damage. Red arrows indicate inhibition, and blue arrows indicate promotion. Green font marks drugs. RANKL, nuclear factor-κB ligand; M-CSF, macrophage colony-stimulating factor; TNF, tumor necrosis factor; IL, interleukin; TGF, transforming growth factor; RF, rheumatoid factor; CCP, cyclic citrullinated peptide; CTLA-4, Cytotoxic T lymphocyte-associated antigen4; Th17, T helper cell type 17; DMARDs, disease modifying antirheumatic drugs; ATRA, All-trans retinoic acid; VIP, Vasoactive intestinal peptide.

The imbalance of Th1/Th2 cells plays a crucial role in the occurrence and development of RA (81). Th1 is primarily characterized by the secretion of high-level γ interferon, and also secrete granulocyte-macrophage colony-stimulating factor, IL-2, TNF-α, etc, to mediate cellular immunity. The characteristic cytokines of Th2 is IL-4, which also secretes IL-5、IL-6、 IL-10、IL-13, etc., to mediate humoral immunity (82, 83). Studies have shown that the joint inflammation in RA is dominated by Th1 (84). The Th1/Th2 ratio increased during RA activity (85), and improving the Th1/Th2 balance positively influences RA therapy. Th1 and Th2 are also derived from CD4^+^T cells induced to differentiate under specific cytokine conditions. Therefore, Tregs-targeted RA therapy may also influence Th1/Th2 balance. The imbalance between anti-inflammatory Tregs and pro-inflammatory Th17 accelerates RA progress (55, 86). Tregs primarily maintain self-tolerance and further suppress autoimmunity, while proinflammatory Th17 induces and propagates inflammation. Studies have found that the number and function of Th17 and Tregs in the peripheral blood of RA patients dramatically change, and the Th17/Tregs ratio increases (66, 87–89). During the pathogenesis of RA, Th17, as a pro-inflammatory helper T cell subset, can induce osteoblast nuclear factor-κB receptor activator ligand (RANKL) expression by upregulating osteoclast formation, leading to bone erosion (90). The initial differentiation of Th17 depends on TGF-β and IL-6, and IL-23 promotes functional maturation of Th17 (90). Interleukin-6-triggered Janus kinase (JAK) 2-STAT3 pathway is required for Th17 development. The STAT3 signaling pathway directly regulates RORγt (91), the master molecule of Th17. IL-17A is a characteristic cytokine of Th17 that is abundantly expressed in arthritic joints. It can directly stimulate RANKL expression and mediate cartilage proteoglycan loss and bone resorption, leading to the destruction of articular cartilage and bone (92). In addition, IL-17A is a potent instigator in the inflammatory response, capable of inducing pro-inflammatory cytokines such as TNF-α and IL-6. This leads to a cascade of inflammation and cell infiltration into local joints, contributing to the hallmark symptoms of arthritis (93, 94). These pro-inflammatory cytokines, including TNF-α and IL-6, along with other inflammatory mediators like IL-17, play a pivotal role in the pathogenesis of RA. They act in additive or synergistic ways, amplifying the inflammatory response and driving the progression of RA (93). This suggests a heightened immune response, with the body’s own defenses turned against itself, leading to the chronic inflammation and joint damage characteristic of RA. In active RA patients, there is an observed elevation in the levels of Th17 cells and IL-17A in peripheral blood. Tregs and Th17 antagonize each other in RA, forming a dynamic balance. The imbalance of this immune balance is a significant cause of RA (66, 95, 96) Therefore, increasing peripheral blood Tregs in RA patients, reducing Th17 or IL-17A levels, and improving the Treg/Th17 cell imbalance may be key to RA treatment (**Figure 2**).

## 4 Tregs-targeted therapies in RA

**Figure 3:**
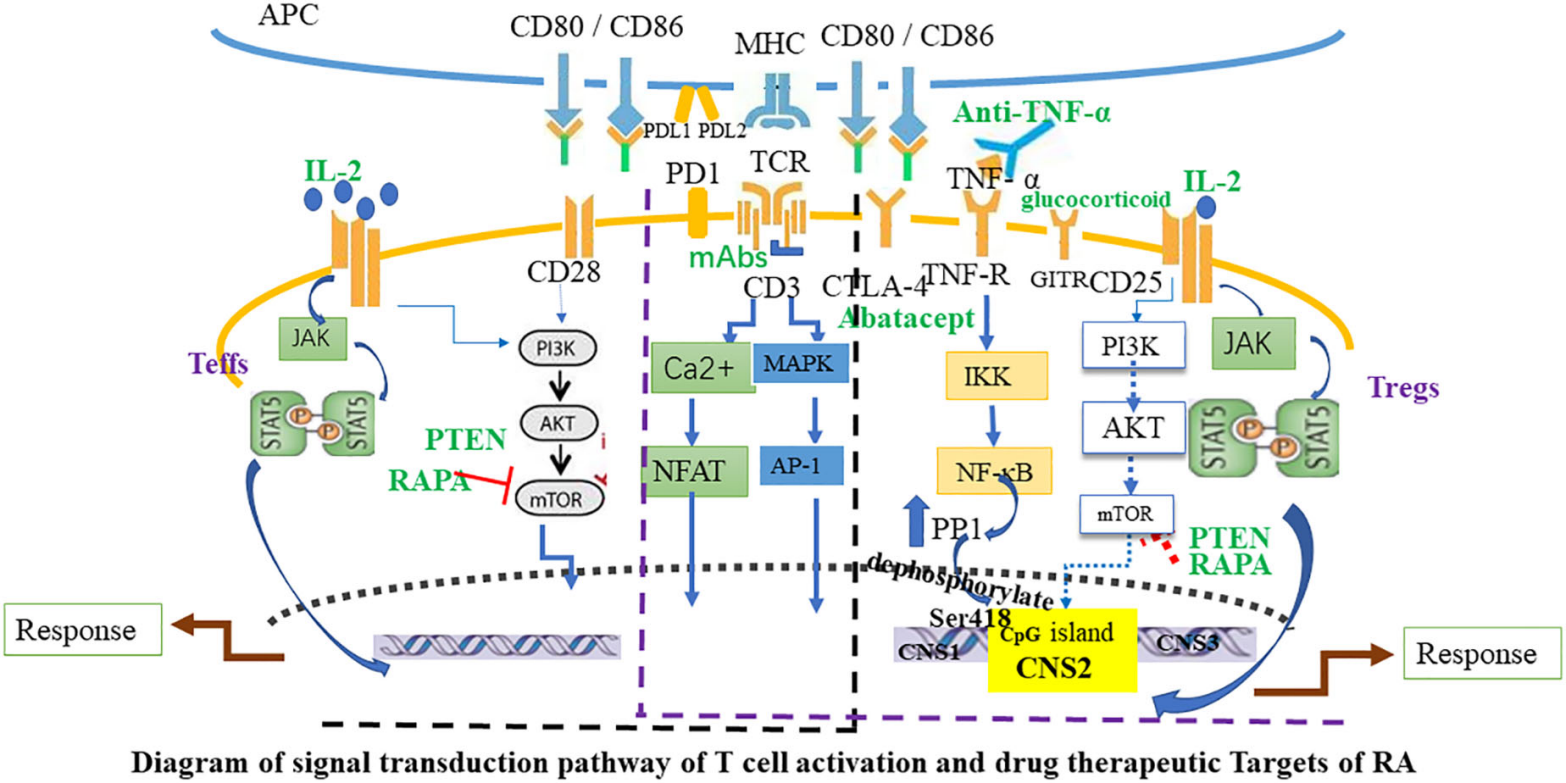
Diagram of signal transduction pathway of T cell activation and drug therapeutic Targets of RA. The activation of Teffs and Tregs is mediated by combined signaling through the TCR and co-stimulatory molecules such as CD28, and through the interleukin-2 receptor (IL-2R). Black font marks molecules expressed by T cells and APC. Green font marks drugs. Blue arrows indicate signaling pathways and red T-bars point to targets of immunosuppressive drugs. APC, antigen-presenting cell; Teffs, effector T cells; JAK, Janus kinase; MAPK, mitogen-activated protein kinase; mTOR, mammalian target of rapamycin; PI3K, phosphoinositide 3-kinase; mAbs, CD3 monoclonal antibodies; CTLA-4, Cytotoxic T lymphocyte-associated antigen 4; PD-1, Programmed cell death protein 1; GITR, glucocorticoid-induced TNFR-related protein; AP-1, activator protein 1; PP1, protein phosphatase 1; PTEN, Phosphatase and tensin homolog; RAPA, Rapamycin.

The search for new, safe, and effective drugs specifically activating Tregs is a significant aspect of cellular immunotherapy. In humans, disease-modifying antirheumatic drugs (DMARDs) are the primary therapeutic agents for RA, reducing synovitis and systemic inflammation, and improving function. Biological agents are employed when arthritis is uncontrolled or when DMARDs induce toxic effects. The therapeutic efficacy of biologics, such as monoclonal antibodies used to neutralize inflammatory cytokines or block cytokine receptors, has been established in halting the progression of rheumatic diseases. However, the risk of infection and high costs limit the prescription of these biological agents. Given the powerful immunosuppressive ability of Tregs, immunotherapy targeting Tregs has become a major direction for the research of autoimmune diseases, including RA. This specialized treatment strives to reinstate physiological self-tolerance by boosting the inherent suppression of pathogenic autoreactive T cells by Tregs, circumventing the side effects typically associated with prolonged immunosuppression or biotherapy. Recently, clinical trials have been initiated to evaluate therapies focused on fostering the expansion of Tregs in autoimmune diseases. In the following sections, we will summarize Treg-targeted therapies in RA (**Figures 2**, **3**).

### 4.1 Glucocorticoids

Glucocorticoids, a frequently used class of drugs, can enhance the presence of Tregs by upregulating Foxp3 expression (97). These drugs can interact with specific receptors present in T cells, leading to a disruption in the communication between TCR signal transduction and the pathways that follow (98). The interaction of glucocorticoids with the exterior of T cells can obstruct the vital interaction between T cells and APCs, a process crucial for an effective immune response. This interaction also downregulates the rolling and adhesion of leukocytes, a key step in immune cell migration, and disrupts the cytoskeleton of T cells, effectively inhibiting their migratory capabilities (99). Moreover, glucocorticoids can shift the balance within the T cell population, favoring the predominance of Th2 cells and Tregs, both of which play crucial roles in immune regulation (100). Notably, patients with autoimmune and atopic disorders, who are treated with glucocorticoids, have demonstrated an increased proportion of Tregs within their T cell subsets (101). Glucocorticoids can also spur the generation of pTregs by boosting the expression of the glucocorticoid-induced leucine zipper (GILZ). This protein further stimulates TGF-β signaling and Foxp3 expression (102). Interestingly, while GILZ appears to be a potent catalyst for Foxp3^+^ expression, the absence of GILZ does not entirely obstruct Foxp3^+^ expression in Tregs. This observation implies that glucocorticoids may enhance Tregs through several, concurrent mechanisms (103). Lilla et al. (104) found that regulatory T cells were less sensitive to glucocorticoid-induced apoptosis than CD4+ T cells and better understood the changes in GC-induced apoptosis-related proteins. Studies have reported that dexamethasone given in the absence of Tregs completely loses its ability to control inflammation, and the Tregs themselves lack glucocorticoid receptors, resulting in rapid loss of therapeutic ability. Glucocorticoids Anti-inflammatory effects are mediated by Foxp3 ^+^ T cell regulation through an MR-342-dependent mechanism (105). In conclusion, glucocorticoids could potentially amplify the prevalence and activity of Tregs. Moreover, they can shape a favorable immune environment for Tregs by adjusting local cytokine expression.

### 4.2 DMARDs

In terms of therapeutic strategies, studies have evaluated the immune responses in mice immunized with ovalbumin and treated with Methotrexate (MTX), Cyclophosphamide (CTX), or a combination of both. Observations have shown that combination therapy of MTX and CTX, as opposed to monotherapy with either drug, induces a bias towards Tregs and inhibits Th17 by interfering with the maturation and antigen-presenting abilities of DCs (106). Upon interaction with this compound, there is a prolonged upregulation of Foxp3, TGF-β, and IL-10 in CD4^+^ cells, an augmentation of Treg suppressive function, and a reduction in IL-17 mRNA (107). In the context of hydroxychloroquine, when this compound is introduced to peripheral blood mononuclear cells from RA patients *in vitro*, there is a decrease in the secretion of IL-17, IL-6, and IL-22 in the culture supernatant (108). Treating early, untreated RA patients with methotrexate can increase the proportion and absolute number of Tregs with high levels of activation markers, indicating an enhancement of their functional capabilities (109). A study evaluated the effect of MTX treatment on the percentage and absolute number of CD4^+^Foxp3^+^ Tregs in the peripheral blood of untreated patients with early-stage RA and found that increases and phenotypic changes in Treg cells were associated with MTX treatment closely (109). MTX-loaded nanoparticles reduced the severity of experimental arthritis models in mice and reciprocally regulated Th17 and Tregs *in vivo*. Nanoparticles are promising therapeutics due to their ability to deliver and release drugs (110). Patients battling autoimmune diseases often necessitate lifelong immunotherapy, a regimen that can result in severe adverse reactions and medicinal side effects. Initiating drug treatment at an early stage can effectively impede the disease’s progression and decrease the rate of advancement. Given the numerous reports on the regenerative capabilities of Tregs, the most effective therapeutic strategy is to induce self-tolerance prior to the onset of significant tissue damage.

### 4.3 Low-dose interleukin-2

Interleukin-2 (IL-2) is crucial for Treg-mediated tolerance and it plays a pivotal role in the generation, expansion, survival, and function of Tregs (111, 112). IL-2 receptor signaling can facilitate the expansion and differentiation of T cells and activate a series of signaling pathways in T cells, including MAPK, PI3K, STAT5, and so on (113). Among them, the STAT5 signaling pathway is an important pathway for Tregs (114). STAT5 activation can directly regulate the expression of Foxp3 and inhibit the methylation of CpG islands in the CNS2 region by binding to Foxp3 promoter and CNS2 region. Simultaneously, Tregs intrinsically express high-affinity CD25 (IL-2 receptor alpha chain) along with unique IL-2-inducing signals and downstream genes (115). They exhibit a preferential response to low doses of IL-2 in comparison to other immune cells (116). The administration of low-dose IL-2 has been significantly effective in reducing disease activity (117). Thus, IL-2 helps tip the immune balance toward regulation rather than inflammation. Research by Scott N. Furlan et al. (118) found that compared to infusing Tregs alone with rapamycin, adding IL-2 *in vivo* to rapamycin supports the logarithmic growth of Tregs and effectively increases the number of Tregs in peripheral blood compartments. Therefore, low-dose IL-2, which is well-tolerated, can induce Tregs and mediate clinical improvements in autoimmune and inflammatory diseases. Employing exogenous low-dose IL-2 to selectively stimulate Tregs appears advantageous for the body, and it holds substantial potential for the treatment of immune diseases. Currently, the development of human anti-IL-2 monoclonal antibodies is underway (119, 120). Scientists have engineered a specific IL-2 monoclonal antibody (mAb), JES6-1, that selectively proliferates Tregs and demonstrates superior disease management in a mouse model of colitis, in comparison to a non-covalently linked complex of IL-2 and JES6-1 (121). In a prospective, open-label, Phase I to IIa study in patients with RA, low-dose IL-2 was shown to be well tolerated and effective, activating and expanding specific Tregs without activating effector T cells, thereby increasing The Treg/Teff ratio (122). Low-dose IL-2 therapy has been tried to treat a variety of autoimmune diseases and is a promising approach for the treatment of RA.

### 4.4 Rapamycin and all-trans retinoic acid

Initially discovered as a powerful antifungal agent derived from Streptomyces hygroscopicus, Rapamycin (RAPA) was subsequently acknowledged for its significant immunosuppressive qualities. It accomplishes this immunosuppression by inhibiting the serine/threonine kinase mTOR, a downstream effector in the phosphatidylinositol 3-kinase (PI3K) and Akt signaling pathway (123, 124). mTOR exerts its effect through two unique complexes, specifically mTOR complex 1 (mTORC1) and mTOR complex 2 (mTORC2). Rapamycin exhibits a stronger inhibitory effect on mTORC1 and only suppresses mTORC2 after prolonged exposure. These mTOR complexes play a crucial role in mediating a myriad of cellular activities. The differentiation of Th1 and Th17 cells from CD4^+^ T cells is directed by mTORC1, while mTORC2 plays an indispensable role in the differentiation of Th2 cells (125). Interestingly, Tregs exhibit resistance to the growth-inhibitory effects mediated by rapamycin. Studies have indicated that rapamycin promotes the proliferation and survival of Tregs, while simultaneously inhibiting the growth, migration, and cytokine production of Th1 and Th17 cells (126–128). Initially, Bruyn et al. (129) found that the clinical efficacy of methotrexate combined with rapamycin analog everolimus in the treatment of RA was much better than that of methotrexate alone. Subsequently, more and more studies have shown that rapamycin is safe and effective in the treatment of RA. The original research group evaluated the effect of rapamycin on CD4 ^+^ CD25 ^+^ Foxp3 ^+^ Tregs in RA patients, suggesting that rapamycin can promote the proliferation of Tregs in low-activity RA patients, thereby balancing Th17/Tregs. Balance of cell ratios increases the likelihood of clinical remission in RA and reduces the risk of disease recurrence (130). Wen et al. (128) found that rapamycin immunomodulatory therapy can not only effectively reverse the reduced Tregs and suppress effector T cells, but also alleviate the clinical symptoms of patients with active RA and reduce the application of immunosuppression. It can be seen that the use of rapamycin can not only control clinical symptoms, but also greatly reduce the dosage of hormones and immunosuppressants in patients. The PI3K-Akt-mTOR signaling axis tightly governs the development, homeostasis, and functionality of Tregs (131). However, due to the widespread expression of the PI3K-Akt-mTOR signaling pathway, the effects of rapamycin can be observed in a variety of immune and non-immune cells. It’s still unclear whether rapamycin selectively inhibits the proliferation of non-Tregs, thereby indirectly promoting the expansion of Foxp3^+^ Tregs (132). Despite a significant increase in Treg levels, rapamycin leads to a transient deterioration of β-cell function in patients with Type 1 Diabetes Mellitus (T1DM) (133). Hence, the negative impacts of this approach in clinical practice cannot be overlooked, and its application in autoimmune diseases requires further investigation. Comparative studies have shown that both rapamycin and all-trans retinoic acid (ATRA) have similar promoting and stabilizing effects during the expansion process of Tregs (134), and under inflammatory conditions, ATRA exhibits superior stability in nTregs compared to rapamycin (135). ATRA enhances the differentiation and stability of iTregs without altering DNA methylation (136). ATRA significantly activates the ERK1/2 signaling pathway, promoting Foxp3 expression (136). DNA methylation at the Foxp3 gene locus plays an important role in the expression and maintenance of Foxp3 in subpopulations (137). In the presence of inflammatory factors, ATRA can inhibit the methylation of the Foxp3 gene in nTregs (135). This may be one of the molecular mechanisms by which ATRA plays a role in diseases of the immune system. Therefore, ATRA can be used as monotherapy or adjuvant therapy for autoimmune diseases.

### 4.5 Biological agents

#### 4.5.1 Anti-TNF agents

In the past 10 years, more and more studies have emphasized the impact of biological agents on Tregs in RA patients. Ehrenstein et al. initially observed that infliximab (a chimeric monoclonal antibody against TNF) treatment increased the proportion of circulating CD4 ^+^ Foxp3^+^ cells and restored the inhibitory activity of CD25^high^ Tregs (138). Studies suggest that the expression of Foxp3 in CD4^+^ lymphocytes escalates in patients treated with etanercept, a fusion protein that functions as a TNF inhibitor (139). Patients treated with TNF-α blockers had a higher proportion of Tregs than patients treated with methotrexate (MTX) (140). Anti-TNF-α treatment increases the Tregs/effector T cell ratio, indicating that it can restore the suppressive function of Tregs (57, 141). Furthermore, Tregs isolated from RA patients who have responded favorably to adalimumab monotherapy seem to display more pronounced suppressive activity (142, 143).However, some studies did not observe any difference in the percentage of Tregs before and after treatment with the human monoclonal antibody adalimumab (50, 144). Some have shown that blocking TNF affects the number and function of Tregs in mouse arthritis models and RA patients (145, 146). Recent studies by Tseng et al. found that TNFR2 is required for Treg development and function under homeostatic and inflammatory conditions. They further verified that TNFR2 is required to prevent Foxp3 promoter DNA methylation, emphasizing the importance of TNFR2. TNFR2 signaling plays an important role in the maintenance of Tregs stability, and TNFR2 deficiency results in a Th17-like phenotype (147). Valencia et al. found that TNF is mediated by TNFRII, which is constitutively expressed by Tregs, and that downregulation of expression results in a reduction of Foxp3 mRNA, directly impairing the inhibitory activity of RA CD25^high^ Tregs (148). The expansion and activation of TNFRII^+^ Tregs may be one of the mechanisms by which anti-TNF drugs control RA inflammation (149). A recent investigation also demonstrated that TNF-α modulates the balance between Tregs and pathogenic Th17 and Th1 cells in the synovium of RA patients via Foxp3 dephosphorylation (72, 148). In RA patients, TNF blocker treatment restored the suppressive activity of Tregs, which was associated with reduced protein phosphatase 1 (PP1) expression and increased Foxp3 phosphorylation in Tregs. The critical phosphorylation site Ser418 in the c-terminal DNA-binding domain of Foxp3 is a critical site for the inhibitory function of Tregs. In RA-derived Tregs, TNF-α amplifies the expression of protein phosphatase 1 (PP1) via the IKK-NF-κB pathway, which results in the dephosphorylation of the crucial Ser418 site of Foxp3. This action subsequently leads to the inactivation of Foxp3, causing damage (150). Interestingly, animal model studies have shown that ntregs require the presence of TNFα to function effectively, but iTregs do not require this stimulation (151). Therefore, TNF-α is a major driver of inflammation in RA patients, and TNF blockers are an effective treatment for RA.

#### 4.5.2 CTLA-4-immunologlobulin-G1

The fusion protein linked with the cytotoxic T-lymphocyte-associated antigen 4 (CTLA-4-Ig), which tactically adjusts the costimulatory signaling between CD28 and CD80/86, appears to function as a biological DMARDs. Notably, this substance has a dual role: it not only engages with T cells, but also has a significant interaction with other cell populations that play a critical role in the development and progression of RA (152). The first description of CTLA-4 abnormalities in RA-deficient Tregs dates back to 2008, when RA patients had lower CTLA-4 levels and higher internalization rates in Tregs than healthy subjects (153). Considering that the forced expression of CTLA-4 on Tregs in the context of RA can rejuvenate their inhibitory function, and conversely, the blockade of CTLA-4 on healthy Tregs can impair their functionality, researchers theorize that CTLA-4 present on RA Tregs could be a prospective therapeutic target for direct manipulation within these cells. The contribution of CTLA4-Ig in the treatment of RA extends beyond merely curtailing T cell activation. One of the protective mechanisms of CTLA4-Ig is to directly inhibit the formation of osteoclasts. In fact, CTLA-4 expressed by normal Tregs binds to CD80/CD86 on dendritic cells, inducing the activation of IDO, and leading to the apoptosis of osteoclast precursors (154). Although anti-CTLA‐4 antibodies have been used in autoimmune therapy, their underlying mechanisms have not been fully elucidated. One study showed that CTLA4-Ig inhibited CIA by modifying CIA mouse DCs and converting them into tolerant DCs, thereby increasing the number of CD4^+^CD25^+^Foxp3^+^ Tregs. This may be a new immune regulatory mechanism of CTLA4-Ig (155). CTLA-4-Ig, in conjunction with TCR ligation, possesses the added capacity to transform naive CD4^+^CD25- T cells into Foxp3^+^Tregs and amplify their population (156). Abatacept is a biological treatment that employs the extracellular domain of the CTLA-4 protein, fused with the Fc portion of IgG1, to safely disrupt the costimulatory signal of APC (157). RA patients who carry the CTLA-4 G polymorphism demonstrated a superior EULAR response and a reduced disease activity rate following treatment with abatacept (158). In the European Union, the use of abatacept has been granted approval for patients with highly active and progressive RA who have not previously undergone treatment with methotrexate (159). However, the exact mechanism of the drug’s efficacy and its effects on the cells that most commonly utilize the CTLA-4 protein (tregs) is unclear and may be conflicting. It has been reported that abatacept has a very complex effect on Treg population number and suppressive function (157). For instance, administering abatacept for RA seems to revive, at least partially, the impaired suppressive action of circulating Tregs (160, 161). However, this finding has not been corroborated by *in vitro* studies involving Tregs from synovial fluid (162). In addition, one study has shown that CTLA-4Ig treatment significantly reduced the proportions of Tregs (163).

#### 4.5.3 IL-6 receptor blocker

IL-6 is a key cytokine determining whether naive T lymphocytes differentiate into Tregs or Th17 cells (164). This understanding has inspired us to delve deeper into the effect of inhibiting IL-6 on the Treg/Th17 cell balance in autoimmune diseases like RA. Evidence from experimental RA models suggests that early intervention with anti-IL-6 receptor antibodies can decrease the prevalence of circulating Th17 cells, thereby mitigating clinical symptoms (165). Consistent with these studies, treatment of early-stage RA with the IL-6 receptor blocker tocilizumab for 3 months resulted in a similar reduction in the number of Th17 (166). This reduction was demonstrated in another study that counted Th17 percentages at 4 months after tocilizumab treatment (57). Other studies did not observe any difference in Th17 percentage 6 months after treatment (167, 168). IL-6 is related to the plasticity of Tregs. Data from RA patients treated with tocilizumab show an increase in Treg numbers after treatment, consistent with a good clinical response (57, 168, 169). IL-6 is a major driver of increased inflammation in RA patients, and tocilizumab has been shown to treat RA patients and alleviate their disease (170–172).

### 4.6 Treg-based cellular therapies

In recent years, cell therapy centered around Tregs has emerged as a novel target for treating RA, garnering widespread attention (173–177). One strategy for Tregs therapy in RA involves isolating Tregs from patients and then expanding them *in vitro*. The goal is to enhance immune suppression by increasing the number of Tregs, thereby preventing excessive immune responses leading to arthritis inflammation. Studies have confirmed that adoptively transferred Tregs in the CIA model quickly appear in synovial tissue after injection, blocking T-cell proliferation and type II collagen-specific proliferation, significantly reducing disease severity and slowing disease progression (178). Transfer of Tregs into T-cell-deficient mice has been shown to increase bone volume (179). While various Tregs populations exist in peripheral blood with predictable functional and phenotypic differences based on cell surface markers (180), their clinical application is limited due to their scarcity, lack of reactivity, and unclear specificity. Successful Tregs transfer therapy for RA patients requires inputting a sufficient number of cells and effectively expanding antigen-specific Tregs without losing their specificity or function. Researchers are exploring methods to enhance the specificity of Tregs through genetic engineering, aiming to improve their inhibitory effect on specific autoantigens. This can be achieved by introducing specific antigen receptors (such as TCRs (181, 182) or chimeric antigen receptors (CARs) (183–185), allowing Tregs to more precisely target and suppress inflammation in the joint area. The adoptive transfer of these antigen-specific Tregs has been shown to be stable *in vivo* and reverse CIA progression by inhibiting CD4+ T cell proliferation and the key inflammatory cytokine TNF-α (186). Compared to non-specific TCR transduction or polyclonal Tregs, TCR Tregs exhibit enhanced inhibitory functions, making them more capable of restoring dominant immune tolerance and completely alleviating targeted autoimmune diseases (187). CAR Tregs have stronger signaling capabilities than TCR Tregs (188). Recently, Safari and colleagues (189) revealed the potential of the clustered regularly interspaced short palindromic repeat (CRISPR) genome editing technique combined with the CRISPR-associated (Cas) 9 system to modify Tregs (189).

In another realm, research is exploring the combination of Tregs therapy with other immune-modulating therapies to enhance efficacy. This may involve the co-application of Tregs with immunomodulatory drugs or other cell therapies, regulating the immune system at multiple levels to achieve a more comprehensive treatment outcome. An ongoing phase I clinical trial (NCT02772679) is investigating the combined administration of IL-2 and polyclonal Tregs. As mentioned earlier, low-dose IL-2 treatment itself has the effect of expanding Tregs *in vivo*. The combined administration of polyclonal Tregs with low-dose IL-2 is expected to enhance the number and function of Tregs after infusion. Immunosuppressive drugs commonly used to treat autoimmune diseases have been shown to dose-dependently reduce Treg proliferation and survival (190, 191). Combining Tregs with other cell therapy methods may produce synergistic effects in the treatment of autoimmune diseases, and such approaches are yet to be explored (192). Adoptive transfer of Tregs, whether used in treating autoimmune diseases or employing a strategy of increasing Tregs, aims not only to correct the balance between Tregs and Teff but also faces challenges. One challenge is maintaining the stability and functionality of Tregs during *in vitro* expansion (193, 194). Research on how to isolate phenotypically stable, highly active, and functionally suppressed Tregs and designing strategies to increase their numbers while maintaining their characteristics is crucial for developing Treg-based therapies for autoimmune diseases. Additionally, factors such as individual differences and potential adverse reactions during the treatment process need to be carefully considered. Therefore, while Tregs therapy brings new therapeutic prospects for RA patients, further in-depth research and clinical trials are needed to validate its safety and effectiveness.

### 4.7 Others

Phosphatase and tensin homolog (PTEN), a tumor suppressor and a phosphatase specific to the 3’ position of phosphatidylinositol 3,4,5-triphosphate (195), has a pivotal role in cell metabolism and motility (196). Research indicates that PTEN exhibits therapeutic potential in CIA rats and has associations with RA (197, 198). PTEN can enhance the stability of Tregs, while its absence can trigger autoinflammatory conditions (199, 200). Systemic administration of PTEN attenuated arthritis severity in CIA mice, and an increased expression of PTEN curtailed T-cell activation and ameliorated the imbalance between Th17 cells and Tregs (200). PTEN operates as an intracellular phosphatase, putting the brakes on the PI3K-Akt-mTOR signaling pathway. Excessive stimulation of this pathway, a scenario observed in PTEN-deficient Tregs, can incite Treg instability due to the hyperactivation of mTORC2 (199, 201). A hallmark of synovial fibroblast activation in RA patients is the downregulation of PTEN expression (202). These studies have shown that PTEN plays a key role in the occurrence and development of immune responses, and it may be used to modify Tregs function. Overexpression of PTEN can inhibit the severity of CIA, thereby reducing the inflammatory response and osteoclast generation. Significantly, the absence of p53 is linked with a reduction in PTEN gene expression, which in turn triggers the phosphorylation of STAT3, thereby exacerbating autoimmune arthritis. Consequently, this discovery implies that PTEN could potentially be harnessed to alter the function of Tregs (200). In addition, studies have shown that TGF-β, vasoactive intestinal peptide (VIP) and immunoglobulin D (Immunoglobulin D, IgD)-Fc-Ig play an important role in targeting Tregs to restore immune tolerance in RA. TGF-β is an important growth factor that promotes Th17 and Treg differentiation. In the context of experimental arthritis, while the local suppression of TGF-β1 signaling can enhance the Th17/Treg equilibrium, it does not yield improvements in joint pathology (203). VIP is a neuropeptide with anti-inflammatory effects and has therapeutic potential in a variety of immune diseases. It is involved in maintaining immune tolerance through a novel mechanism that induces the generation of Tregs (204). IgD-fc-ig selectively interferes with the interaction between IgD and IgDR, putting a halt to the abnormal proliferation and activation of T cells provoked by IgD. It corrects the imbalance between Th17 and Tregs, lessens the production of inflammatory cytokines (205). The composition of the gut microbiota in patients with RA undergoes changes, potentially exacerbating immune dysregulation (206, 207). Fecal microbiota transplantation, involving the introduction of specific bacteria, may become an adjunctive approach in treating RA. Short-chain fatty acids, produced by certain gut bacteria, have demonstrated anti-inflammatory effects (208).They can upregulate anti-inflammatory genes in dendritic cells, enhance histone acetylation of the Foxp3 locus, and increase the stability of the Foxp3 transcription factor (209). Other molecules produced by bacteria, such as Bifidobacterium’s polysaccharide A, can induce the generation of Tregs (210). Due to safety concerns, fecal transplantation therapy has not yet been approved clinically. However, once this therapy becomes viable, it could be considered as a potential Treg-promoting strategy, synergizing with Tregs-based therapies or other methods promoting Tregs to enhance their therapeutic efficacy.

## 5 Summary

The maintenance of self-immune tolerance is greatly reliant on Tregs. A decline in the number and/or function of Tregs in RA patients indicates that abnormalities in Tregs are intimately associated with the progression of RA. The transfer of Tregs and preservation of Tregs functions present promising strategies to mitigate joint inflammation in RA patients. CAR-Tregs could potentially serve as a useful tool for treating, or even curing, autoimmune diseases. However, the regulatory mechanisms of Tregs are not yet fully comprehended and warrant further investigation. There is a pressing need to bolster research in clinical investigations and related animal experiments to explore the immune mechanism of Tregs in the pathogenesis of RA. It is anticipated that the scope for future treatment of RA will expand considerably.

## Author contributions

JZ: Writing – original draft. HJL: Writing – review & editing. YC: Funding acquisition, Writing – review & editing. HL: Writing – review & editing. SZ: Writing – review & editing. QX: Funding acquisition, Writing – review & editing. GY: Writing – review & editing.
